# Draft Genome Sequence of Bacillus anthracis Strain Sterne 09RA8929

**DOI:** 10.1128/MRA.00972-18

**Published:** 2018-10-11

**Authors:** Anne Busch, Mandy Carolina Elschner, Daniela Jacob, Roland Grunow, Herbert Tomaso

**Affiliations:** a Friedrich-Loeffler-Institut, Institute of Bacterial Infections and Zoonoses, Jena, Germany; b Centre for Biological Threats and Special Pathogens, Robert Koch Institute, Highly Pathogenic Microorganisms (ZBS 2), Berlin, Germany; University of California, Riverside

## Abstract

A Bacillus anthracis vaccine strain (Sterne), used as an attenuated laboratory comparative strain, was sequenced and analyzed. A comparison to assemblies of B. anthracis strain Sterne (NZ_CP009541 and NZ_CP009540) was performed. The lack of the pX02 plasmid and pX01 in approximately five copies was confirmed.

## Abstract (precis)

A Bacillus anthracis vaccine strain (Sterne), used as an attenuated laboratory comparative strain, was sequenced and analyzed. A comparison to assemblies of B. anthracis strain Sterne (NZ_CP009541 and NZ_CP009540) was performed.

## ANNOUNCEMENT

Bacillus anthracis, a Gram-positive, spore-forming bacterium, is the etiological agent of anthrax (1, 2), a zoonotic disease that can infect humans directly or through products. To prevent anxtrax infections in livestock, vaccines comprising live B. anthracis strains (pX01^+^ [pX01 positive]; pX02^−^ [pX02 negative]) for animals are available (3). We sequenced and analyzed the B. anthracis strain Sterne 09RA8929 at the Friedrich-Loeffler-Institut (FLI) to use it as a laboratory comparative reference strain.

The strain was provided from a repository located at the Robert Koch Institute within the framework of the European Union–funded projects EQADeBa and EMERGE (grant agreement 677066) after two passages on Columbia sheep blood agar plates (Oxoid, Wesel, Germany). At the FLI, the isolate was cultivated for diagnostic purposes and added to the repository. The culture for DNA extraction was initiated from a single colony. DNA was extracted from bacterial cells that were grown in cell culture flasks for 24 h. DNA purification was performed using a Genomic-tip 100/G instrument and a genomic DNA buffer kit (Qiagen, Hilden, Germany) following the manufacturer’s instructions. Genome sequencing was carried out using Illumina DNA sequencing at GATC Biotech (Germany) with the library protocol adapted and validated for use with the HiSeq 2500 platform. A paired-end library was constructed with a target insert size of 300 bp, which resulted in 6 million reads. A total of 12 million (151-bp) sequences, with an average Phred score of >38, were generated. The sequences were assembled using SPAdes version 3.9.1 in Bayes Hammer mode, to minimize mismatches and short indels (--careful) (4) and filtered for contamination with Kraken (5). Subsequently, annotation was performed with Prokka using the standard settings (6). The assembly resulted in 33 contigs with an *N*_50_ value of 1,162,008 bp and a total sequence length containing 5,366,275 bp. The annotation predicted 12 RNAs, 5,639 coding DNA sequences (CDSs), 79 tRNAs, and 1 tmRNA. A phylogenetic analysis of the 16S rRNA sequences with Mole-BLAST and PhyloPhlAn (7) produced consistent results. From the phylogeny determined with PhyloPhlAn, a differentiation within the Bacillus genus was possible, although the coding sequences of the plasmids and highly variable sequences are excluded with this method. This bioinformatics approach complements the methods of single-nucleotide polymorphism typing and multilocus variable-number tandem-repeat analysis.

After quality checking, trimming, and merging with BBDuk, and removal of duplicates, the reads were mapped to other genome sequences in GenBank (accession numbers NZ_CP009540, NZ_CP009541, and NZ_CP010794) with the Bowtie2 module included in Geneious (7), and variant-calling files were generated. Variants are reported with a minimum variant frequency of 0.9, a minimum *P* value of 10e-60, and a minimum strand bias *P* value of 10e-5, and supported by at least five reads. A 37-fold mean coverage of the chromosome was reported with 5 million bases showing 99.99% identity with the reference sequence (5,227,150 of 5,227,496). For the chromosome, 88 variants were called, and 36 variants were predicted to affect known proteins. The plasmid pX01 yielded a 161-fold mean coverage of 181,803 bases with 99.8% identity to the reference sequence (181,233 of 181,624). For the plasmid, 13 variants were called; seven of these affected known proteins (Table 1). With this described method and data, future B. anthracis strains can be monitored. The average chromosome/plasmid coverage ratio suggests that the plasmid pX01 is unusually represented with approximately five copies per cell. It has been reported that sequencing coverage provides exact estimates for the plasmid copy number (8). Since no mapping occurred on one of the pX02 reference sequences (GenBank accession number NZ_CP010794), the existence of pX02 could be excluded.

**TABLE 1:** Features of the genome of B. anthracis Sterne 09RA8929, including variants to the chromosome and plasmid sequences^*a*^

RefSeq no.	Nucleotide position	Reference	Alteration	Variation type	CDS type^*b*^	Translational effect
NZ_CP009541	345217	C	T	SNP^*c*^ (transition)	Hypothetical protein	None
NZ_CP009541	345220	CGG	TGC	Substitution	Hypothetical protein	Substitution
NZ_CP009541	345226	T	G	SNP (transversion)	Hypothetical protein	None
NZ_CP009541	959629	G	T	SNP (transversion)	Transposase	Substitution
NZ_CP009541	959936	C	T	SNP (transition)	Transposase	Substitution
NZ_CP009541	1273892	T	TA	Insertion (tandem repeat)	His B	Frameshift
NZ_CP009541	1291383	A	G	SNP (transition)	x	x
NZ_CP009541	1291518	C	A	SNP (transversion)	x	x
NZ_CP009541	1291524	T	C	SNP (transition)	x	x
NZ_CP009541	1291568	T	G	SNP (transversion)	x	x
NZ_CP009541	1291570	A	G	SNP (transition)	x	x
NZ_CP009541	1291573	C	T	SNP (transition)	x	x
NZ_CP009541	1291606	C	T	SNP (transition)	x	x
NZ_CP009541	1291608	A	G	SNP (transition)	x	x
NZ_CP009541	1291642	CT	C	Deletion (tandem repeat)	x	x
NZ_CP009541	1442326	T	TG	Insertion (tandem repeat)	Hypothetical protein	Frameshift
NZ_CP009541	1442340	A	G	SNP (transition)	Hypothetical protein	Substitution
NZ_CP009541	1442346	A	G	SNP (transition)	Hypothetical protein	Substitution
NZ_CP009541	1442349	A	G	SNP (transition)	Hypothetical protein	Substitution
NZ_CP009541	1442358	G	A	SNP (transition)	Hypothetical protein	Substitution
NZ_CP009541	1442367	G	A	SNP (transition)	Hypothetical protein	Substitution
NZ_CP009541	1442379	T	TG	Insertion (tandem repeat)	Hypothetical protein	Frameshift
NZ_CP009541	1442465	G	A	SNP (transition)	Hypothetical protein	Substitution
NZ_CP009541	1442471	G	A	SNP (transition)	Hypothetical protein	Substitution
NZ_CP009541	1518305	G	GT	Insertion (tandem repeat)	Isocitratelyase	Frameshift
NZ_CP009541	1602248	G	GT	Insertion (tandem repeat)	x	x
NZ_CP009541	1644562	T	TA	Insertion (tandem repeat)	x	x
NZ_CP009541	1820884	G	GA	Insertion (tandem repeat)	Quinolone resistance protein	Frameshift
NZ_CP009541	1835869	A	AT	Insertion (tandem repeat)	x	x
NZ_CP009541	1911501	A	AT	Insertion (tandem repeat)	Amino acid permease	Frameshift
NZ_CP009541	2082647	A	AT	Insertion (tandem repeat)	Thioredoxin	Frameshift
NZ_CP009541	2218818	C	CT	Insertion (tandem repeat)	x	x
NZ_CP009541	2270478	G	GA	Insertion (tandem repeat)	Alkyl-hydroperoxide reductase subunit F	Frameshift
NZ_CP009541	2340581	G	GA	Insertion (tandem repeat)	Hypothetical protein	Frameshift
NZ_CP009541	2442080	TA	T	Deletion (tandem repeat)	Bcr/CflA family drug resistance efflux transporter	Frameshift
NZ_CP009541	2474920	TC	T	Deletion (tandem repeat)	x	x
NZ_CP009541	2474929	TA	T	Deletion	x	x
NZ_CP009541	2611141	T	TA	Insertion (tandem repeat)	Hypothetical protein	Frameshift
NZ_CP009541	2737890	A	AT	Insertion (tandem repeat)	x	x
NZ_CP009541	2782850	G	GA	Insertion (tandem repeat)	Acyl-CoA-dehydrogenase	Frameshift
NZ_CP009541	2823935	G	GT	Insertion (tandem repeat)	NADH-quinoneoxido reductase subunit H	Frameshift
NZ_CP009541	2906141	A	G	SNP (transition)	Transposase CDS	Substitution
NZ_CP009541	2906932	G	A	SNP (transition)	Transposase CDS	Substitution
NZ_CP009541	2923257	T	TA	Insertion (tandem repeat)	Chitooligosaccharide deacetylase	Frameshift
NZ_CP009541	2923451	C	CT	Insertion (tandem repeat)	x	x
NZ_CP009541	2946626	T	TA	Insertion (tandem repeat)	x	x
NZ_CP009541	3004271	T	TA	Insertion (tandem repeat)	x	x
NZ_CP009541	3011141	G	GT	Insertion (tandem repeat)	x	x
NZ_CP009541	3128843	G	GA	Insertion (tandem repeat)	Lipoylsynthase	Frameshift
NZ_CP009541	3169582	A	AT	Insertion (tandem repeat)	Hypothetical protein	Frameshift
NZ_CP009541	3169588	A	AT	Insertion (tandem repeat)	Hypothetical protein	Frameshift
NZ_CP009541	3201261	A	AT	Insertion (tandem repeat)	x	x
NZ_CP009541	3201734	TA	T	Deletion	x	x
NZ_CP009541	3201748	G	A	SNP (transition)	x	x
NZ_CP009541	3330387	TC	CT	Substitution	Spore germination protein GerIA	Substitution
NZ_CP009541	3330410	C	T	SNP (transition)	Spore germination protein GerIA	Substitution
NZ_CP009541	3330416	TTC	GAA	Substitution	Spore germination protein GerIA	Substitution
NZ_CP009541	3330421	G	T	SNP (transversion)	Spore germination protein GerIA	Substitution
NZ_CP009541	3330424	GC	AT	Substitution	Spore germination protein GerIA	Substitution
NZ_CP009541	3330430	C	T	SNP (transition)	Spore germination protein GerIA	None
NZ_CP009541	3361291	C	CT	Insertion (tandem repeat)	x	x
NZ_CP009541	3364114	A	G	SNP (transition)	x	x
NZ_CP009541	3389585	G	GA	Insertion (tandem repeat)	Hypothetical protein	Frameshift
NZ_CP009541	3491916	C	CA	Insertion (tandem repeat)	DNA polymerase/3'-5'-exonuclease PolX	Frameshift
NZ_CP009541	3537995	T	TA	Insertion (tandem repeat)	ABC transporter ATP-binding protein	Frameshift
NZ_CP009541	3647307	G	GT	Insertion (tandem repeat)	Rsf A family transcriptional regulator	Frameshift
NZ_CP009541	3647932	G	GT	Insertion (tandem repeat)	Recombinase Rar A	Frameshift
NZ_CP009541	3695170	A	AT	Insertion (tandem repeat)	GNAT family *N*-acetyltransferase	Frameshift
NZ_CP009541	3726079	T	TA	Insertion (tandem repeat)	Molecular chaperone DnaJ	Frameshift
NZ_CP009541	3744057	G	GA	Insertion (tandem repeat)	RNA polymerase sigma factor RpoD	Frameshift
NZ_CP009541	3873967	C	CT	Insertion (tandem repeat)	Pyrroline-5-carboxylatereductase	Frameshift
NZ_CP009541	3951268	GA	G	Deletion	phospho-enol-pyruvate protein phosphotransferase	Frameshift
NZ_CP009541	4137011	C	CT	Insertion (tandem repeat)	x	x
NZ_CP009541	4328378	C	CT	Insertion (tandem repeat)	x	x
NZ_CP009541	4391405	C	CA	Insertion (tandem repeat)	Holin	Frameshift
NZ_CP009541	4422132	C	A	SNP (transversion)	x	x
NZ_CP009541	4766848	C	CT	Insertion (tandem repeat)	x	x
NZ_CP009541	4828439	T	TA	Insertion (tandem repeat)	x	x
NZ_CP009541	4994978	T	TA	Insertion (tandem repeat)	Multidrug MFS^*d*^ transporter	Frameshift
NZ_CP009541	5006878	G	C	SNP (transversion)	Transposase	Substitution
NZ_CP009541	5007511	T	C	SNP (transition)	Transposase	Extension
NZ_CP009541	5007520	A	C	SNP (transversion)	x	x
NZ_CP009541	5007525	G	A	SNP (transition)	x	x
NZ_CP009541	5007546	A	C	SNP (transversion)	x	x
NZ_CP009541	5007607	A	C	SNP (transversion)	x	x
NZ_CP009541	5037843	T	G	SNP (transversion)	ABC transporter permease	Substitution
NZ_CP009541	5116913	CG	TA	Substitution	Enterotoxin CDS	Substitution
NZ_CP009541	5116917	A	G	SNP (transition)	Enterotoxin CDS	None
NZ_CP009540	1	G	A	SNP (transition)	Hypothetical protein	Substitution
NZ_CP009540	2	A	G	SNP (transition)	Hypothetical protein	None
NZ_CP009540	4	A	AACA	Insertion	Hypothetical protein	Insertion
NZ_CP009540	13115	T	TA	Insertion (tandem repeat)	Hypothetical protein	Frameshift
NZ_CP009540	17095	C	CT	Insertion (tandem repeat)	x	x
NZ_CP009540	20289	A	AT	Insertion (tandem repeat)	x	x
NZ_CP009540	65111	G	GA	Insertion (tandem repeat)	Hypothetical protein	Frameshift
NZ_CP009540	87185	T	TA	Insertion (tandem repeat)	PrgI family protein	Frameshift
NZ_CP009540	126302	A	AT	Insertion (tandem repeat)	IS4 family transposase	Frameshift
NZ_CP009540	126914	G	GT	Insertion (tandem repeat)	X	x
NZ_CP009540	128396	C	CT	Insertion (tandem repeat)	IS4 family transposase	Frameshift
NZ_CP009540	162415	C	CT	Insertion (tandem repeat)	x	x
NZ_CP009540	181623	G	A	SNP (transition)	Hypothetical protein	None

a The GenBank accession number for the chromosome sequence is NZ_CP009541 and for the plasmid sequence is NZ_CP009540.

b CoA, coenzyme A; x, no CDS, and thus no translational effect, was predicted.

c SNP, single nucleotide polymorphism.

d MFS, major facilitator superfamily.

### Data availability.

The genome sequence of B. anthracis strain Sterne 09RA8929 has been deposited in NCBI GenBank under BioSample number SAMN09635715 and BioProject number PRJNA422985. Raw data have been submitted to the Sequence Read Archive under accession number SRP159486.
